# From exceptional to common presence: Italian women in twentieth-century life sciences

**DOI:** 10.1007/s40656-022-00550-7

**Published:** 2022-12-02

**Authors:** Ariane Dröscher

**Affiliations:** Dip. di Biologia, via del Proconsolo, 12, 50122 Florence, Italy

**Keywords:** Gender, Women biologists, Early genetics, Hydrobiology, Entomology, Rina Monti

## Abstract

This essay surveys the situation of Italian women life scientists from the late nineteenth to the mid-twentieth century. It follows the path that took women from being an exceptional presence to becoming a common, yet not equal, presence in the Italian science departments. Very different proportions of women occupied the three ranks in the academic hierarchy—students, research staff and professors. From the late nineteenth century onwards, women started to enrol in Italian universities. Initially, the second most popular department among female students—outdone only by the humanities—was that of mathematics, physics and natural sciences. Concerning women among research staff, a brief statistical analysis reveals the growing proportion of the female workforce in academic institutions and brings into view poorly known female assistants and technicians. The most difficult career step for women was to gain a tenured university position. A comparison between bacteriologist Giuseppina Cattani’s ‘failure’ to gain such a position and the ultimately successful strategy of zoologist and limnologist Rina Monti, who became one of the very first female university professors in Europe, illustrates the opportunities as well as the obstacles women naturalists encountered on the way into the academia. These experiences and those of others show that well into the twentieth century the support of powerful male mentors continued to be indispensable for women scientists. Positions in peripheral institutes or specializations in emerging research fields, in particular hydrobiology, entomology and cytogenetics, provided opportunities for Italian women to work their way up to professorships.

## Introduction

At first glance, the history of the relationship between women and science in Italy appears contradictory. Narratives of Italian machismo and Italy’s delay in granting women equal rights stand in contrast with an extraordinary and long tradition of Italian women participating in scientific research. More than a century ago, John Augustine Zahm (writing under the pseudonym H. J. Mozans) emphasised how exceptional the situation in Italy was. The University of Bologna, for instance, had female lecturers in law as early as the thirteenth century, in medicine in the fourteenth century, and stands out by virtue of the high number of women who graduated and were hired during the eighteenth century (Mozans, 1913, pp. 53). In 1678, Elena Lucrezia Cornaro Piscopia (1646–1684) obtained a university degree in philosophy from Padua. The eighteenth century saw even more female professors, with mathematician Maria Gaetana Agnesi (1718–1799), physicist Laura Bassi (1711–1778), and anatomist Anna Morandi Manzolini (1714–1774) teaching contemporaneously at the University of Bologna, later joined by linguist Clotilde Tambroni (1758–1817). Looking beyond the walls of academia and into the private laboratories of Renaissance and early modern courts, monasteries, salons and shops reveals even more Italian women who actively participated in the scientific enterprise (see e.g. Ray, 2015).

Regarding the origins of the unusual role of female scholars in Italy, Zahm recalled the spirit of independence of Ancient Roman women and their demand to be treated as intellectually equal. In Italy, women were thus given more space and opportunity to demonstrate their skills and achievements in all fields of intellectual and artistic expression (Mozans, 1913, pp. 80–81). Rebecca Messbarger (2002) confirms that numerous elite Italian women of the Enlightenment asserted their rights to participation and visibility in the Republic of Letters, yet she paints a more complex picture of contemporary disputes around the education and intellectual skills of women and highlights the role of male authorities. Italy certainly saw more women able to display their intellectual, discursive, cultural and managerial skills in public arenas such as academies and universities than any other country at that time, yet this was anything but a general right. On the contrary, female scientists often clashed with the activities of their male counterparts, which aimed to contain the female presence within certain boundaries. Indeed, other studies of the situation of women scientists in Italy’s Enlightenment (e.g. Berti Logan, 1994; Cavazza, 2014; Findlen, 1995; Findlen et al., 2009; Knott & Taylor, 2005) mostly confirm the view that these pioneer women were able to carve out careers because they were protected and supported by influential men, mostly fathers, brothers, and husbands. Nevertheless, they played an important role as a vanguard, models for other women, and as tokens that proved wrong those who doubted that women were suited to research and teaching.

The Italian women scientists of the nineteenth and twentieth centuries are less well known, even though they too were strikingly active, in particular in the life sciences. During the second half of the nineteenth century, Italian women researchers were the most prolific of all European women scientists, except the British, producing 27 percent of all scientific publications authored by women (Creese, 2004, p. 209). Of these papers, a 65 percent treated biomedical topics, including some 16 percent of botanical papers (ibid., p. 191). Alongside skilled amateur scientists such as Tuscan marchesa Marianna Panciatichi Paulucci (1835–1919) and Roman countess Elisabetta Fiorini Mazzanti (1799–1879), who were both internationally active and widely known for their excellent naturalist skills, younger women entered the scene who wanted to become professional scientists and pursue university careers.

Recently, several new collections of biographies of ‘excellent Italian women’ have appeared (Babini & Simili, 2007; Crucitti & Bubbico, 2020; Strickland, 2010). More instructive than these lists of individual cases are the systematic inquiries provided by several notable publications at the national level (Focaccia, 2012; Linguerri, 2012) and for specific localities, such as Catania (Branciforte, 2001), Florence (Soldani, 2010), Padua (Martini & Sorba, 2021), Rome (Favina, 2020), Turin (Luciano & Roero, 2008) and Venice (De Rossi, 2005). Yet, much research remains to be done. The volumes just mentioned are almost exclusively biographical in character. It is certainly indispensable to know who the women were that embarked onto scientific careers. However, an assessment of the role of women in twentieth-century Italian biological and biomedical research requires the integration of at least three perspectives.

Firstly, a historical-statistical approach that aims to embed prosopographic data into specific historical contexts and to extend such data by means of systematic surveys to estimate numbers and quantitative trends with respect to women scientists and their research output in comparison to male colleagues and woman researchers in other countries. Investigations of the historical context have shown that women scientists faced a variety of obstacles or enjoyed opportunities, owing to, for instance, legal arrangements, local customs, nepotism and the level of support they received from families and mentors (Ascenzi & Sani, 2020). The statistical approach has already produced precious insights and data that enable geographic comparisons (Cammelli & Di Francia, 1998; Dröscher, 2017; Govoni, 2006, 2009; Norsa, 1902; Paoli, 2011; Ravà, 1902). Moreover, statistics is a tool to grasp the nature of twentieth-century science, characterized as it was by big science and mass universities. Assessing the role of women in twentieth-century science therefore requires a different approach than inquiries into the early modern period or the Enlightenment. Besides, in assessing the role of women in twentieth-century biological sciences, ‘minor characters’ and women who ‘failed’ to pursue an academic career are as illuminating as those who were successful.

Secondly, a qualitative investigation of the research carried out by women. This inquiry points directly at the heart of the question if women are adequate for science. This approach should not limit itself to merely listing women’s research topics, but rather investigate their contribution to the contemporary biological science in general and to their specific field of research in particular. This question is certainly the most interesting for historians of science, but also the most difficult to answer conclusively. A growing number of valuable papers focus on the research of some outstanding women such as Rita Levi Montalcini (1909–2012) or Maria Montessori (1870–1952), but many others remain almost completely unknown. It is time “to seek out the lost and buried women heroes of science” (Kass-Simon & Farnes, 1990, p. xii), yet avoiding the other extreme of unconditional celebration.

Thirdly, an institutional-legal inquiry is essential to complete the picture and to gauge the opportunities and barriers that women encountered on the way into academia. In the course of the nineteenth and twentieth centuries, the nature of science changed considerably. One decisive aspect was that a university degree became an indispensable prerequisite for obtaining teaching and research positions. Therefore, from the late nineteenth century, although female amateur scientists continued to exist and to contribute to the general scientific enterprise, the battle to obtain access to the centres of higher education and acknowledgement in the form of graduations and degrees came to the fore in Italy, as it did in other countries (Paoli, 2011; Pesci, 1989; Pironi, 2020; Soldani, 1989). On the other hand, the spectrum of institutions where one could pursue a professional career in science grew steadily in number and in variety. As we will see, many women took advantage of the foundation of smaller institutes distant from the urban universities.

## Pioneer women: transforming an exceptional into a common presence

The comparatively favourable place Italian women enjoyed in the world of science appears to have persisted to the mid-nineteenth century. Cieślak-Golonka and Morten (2000, p. 68) observe that “A university education was off limits to women almost everywhere in Europe—with one exception: Italy”. In fact, in contrast to many other countries, in Italy women were not officially excluded from universities. The text of the educational law, the *legge Casati*, which extended to the entire kingdom after the Italian unification in 1860–1861, was written in male plural, a form which includes both genders. In 1876, Article 8 of the General University Regulation (*Regolamento generale universitario*) even explicitly addressed women by stating that «Women can enroll into the register of students and auditors (*uditori*), when they provide the required documents or equivalent titles». However promising the legal framework, the actual situation was different, and initially only few women enrolled. The principal impediment was the scarcity of women in secondary schools, the educational level that provided the prerequisite documents to access university. Nevertheless, some women made the leap. The first female graduate of the Kingdom of Italy, the Russian Ernestina Paper (1846–1926), obtained her medical degree in Florence in 1877. The first graduations of women in the departments of mathematics, physics and natural sciences took place in Rome four years later. Both Evangelina Bottero (1859–1950) and Carolina Magistrelli (1858–1939) later taught at the Institute of Female Higher Education in Rome (Govoni, 2007). Up to 1900, a total of 224 women graduated in Italy, 48 of whom had chosen the science departments: 25 in natural sciences, 19 in mathematics, two in physics and two in chemistry (Ravà, 1902). During the period 1904–1911 another 22 women (out of a total of 198 female graduates) obtained a degree in a science discipline (Govoni, 2006, 2009; Paoli, 2011). Courses in science were at all times preferred over medical, juridical, agrarian and engineering courses, yet never as popular as the enrolment in courses in the humanities (Cammelli & Di Francia, 1998, pp. 34–38). A look at a specific university shows that there were 367 women graduates from the science department of the University of Bologna between 1894 and 1934 (Giumanini, 2004). Initially, women only graduated sporadically, but from 1903 onwards at least one woman completed her studies every year. Hence, with respect to the presence of female students in Italian science departments, by the 1900s it was common to see women in the lecture halls.

Women found it more difficult to obtain professional research positions. My complete list of the personell of all Italian science faculties (Dröscher, 2013) provides an insight into the world of visible and invisible research associates. During the period 1860–1915, 45 women were formally employed as assistants or technicians in Italian science departments. These positions were certainly regarded as minor, but they were salaried and therefore relatively privileged, compared to the great number of unpaid (and unregistered) associates. The first female technician was Maddalena Lisa Mussino (1805–1869), who worked as an artist at the Botanical Garden of Turin from 1838 to 1868. The second was Clelia Bonomi (b. 1859), who followed her father Luigi Bonomi as a technician at the Zoological Institute of Turin in 1883 and worked there for over thirty years. From the mid-1890s, we can observe a slow but steady increase in the number of female assistants or technicians from four (1894) to nine (1902), ten (1909), fourteen (1912) and twenty-four (1915). Twenty-four is a remarkable number, yet still only about 8 percent of the total number of assistants and technicians in Italy’s science departments in 1915. On the other hand, during the same period, the much bigger medical-surgical departments employed only twenty-three (1.1%) female assistants and technicians. The main science departments that provided women with paid positions in their institutes and laboratories were Turin (ten), Rome (eight) and Pavia (six). Five women obtained formal posts in Bologna, four in Naples, three in Florence, two in Catania, Genoa, Palermo and Padua, and one in Modena, Pisa, Sassari and Siena. No women were formally employed by the science departments of Cagliari, Messina, Parma or any of the small municipal universities (Dröscher, 2013, pp. 50–52). Contrary to what happened in England, where women were explicitly hired as helps (Hartley & Tansey, 2015), several Italian female technicians had graduated and were waiting, just like their male colleagues, for assistant positions to become vacant. Examples are Maria Bakunin (1870–1956) and Elisa Norsa (1868–1939), who both published several papers during their years as ‘preparators’.

The disciplines with the highest share of female assistants and technicians were zoology and comparative anatomy (with a total of seventeen women, three of whom worked in Ercole Giacomini’s institute in Bologna and four in that of Giovanni Battista Grassi in Rome), followed by botany (seven), physics (four), astronomy (three, all in Turin), anthropology (two), chemistry (two), geology (two), mineralogy (two), meteorology (one), terrestrial physics (one) and physical geography (one). Five women worked in the mathematical sciences. The average term of employment of women in the science departments was 5.4 years, almost twice as long as their female colleagues working in the medical departments, who held their positions for an average time of 2.8 years. Hence, at the beginning of the twentieth century, in line with the situation of female graduates, women had become a minor but visible and constant presence inside many Italian institutes dedicated to the natural, physical and mathematical sciences.

The number of opportunities for a professional career in research became even scarcer if we look at the higher levels of university ranks. Most female research assistants left university after a few years and a couple of publications, and became teachers at secondary schools. Until 1915, only three women became private lecturers (*liberi docenti*)—Maria Bakunin in Naples (holding courses in general chemistry), Maria Montessori in Rome (anthropology) and Rina Monti (1871–1937) in Pavia (comparative anatomy). Two of them succeeded in making the final step to become tenured university professors: Monti became associate professor in 1908 and two years later obtained tenure in Sassari, the smallest of all Italian public universities, and Bakunin became associate professor of chemistry at the Neapolitan Polytechnic in 1912 and obtained tenure in 1917. At the time, only three (0.2%) women taught as private lecturers in Italy’s medical departments: Virginia Angiola Borrino (1880–1965) offered courses in pediatrics in Turin, Giuseppina Cattani (1859–1914) in general pathology in Turin and Bologna, and Maria Masini in criminal anthropology in Genoa. Only Borrino became a tenured professor (in Sienna), yet lost this position after few years due to formalities. Hence, female university professors continued to be the exception in Italy well into the 1920s.

## Reasons for the ongoing rarity of women in higher university ranks

To understand why there continued to be a dearth of women among the higher university ranks, it is worth focusing on the appointment of Rina Monti as well as Giuseppina Cattani’s failure to continue in her university career. Both Cattani and Monti got excellent marks during their studies and won international praise and prizes for their early researches. Both never seriously contemplated any alternative to an academic career (Dröscher, 2007). In 1887, Cattani was the first woman in the Kingdom of Italy to obtain a private lecturship (*libera docenza*) in medicine, first in Turin and two years later in Bologna. She held courses in bacteriology, which had only very recently been introduced as a taught subject in Paris and Berlin (Passione, 2007, pp. 19–20). Her publication output was prolific, with thirty-seven scientific papers between 1884 and 1893. Nevertheless, Cattani was passed over in three competitions for professorships. In 1897, she accepted a position as head of a laboratory for radiology, morbid anatomy and bacteriology at the hospital of her native town of Imola, near Bologna. Rina Monti was more perseverant. In 1900, she became the first female private lecturer at an Italian science department. By then, she had published twenty-four papers, and during the subsequent period 1901–1907 another twenty-one, many of them in foreign journals. However, it took seven appointment competitions, in which her work was initially praised and then criticized with increasing harshness. In 1908, she was finally appointed to become a temporary professor at the small Sardinian University of Sassari. Three years later, she was promoted to a tenured professorship. In 1915, she moved to the prestigious chair of Zoology in Pavia, and in 1924 to the newly founded University of Milan.

What were the main obstacles that thwarted or encumbered the careers of these pioneer women? Both were lone fighters. As in other Western countries, Italian feminists created women’s associations beginning in the late twentieth century. Maria Montessori, for instance, campaigned for the *Società per la Donna*, or *Society for the Woman*, and the German nurse Anna Fraentzel (1878–1858), who assisted her husband Angelo Celli (1857–1914) in his famous studies of malaria, became president of the Roman section of the *Unione femminile nazionale*. But the most active women in these associations were school teachers, whose aim was to improve primary and secondary schooling. A specific association endorsing female academics came into being much later. Around 1923, Isabella Grassi (1886–1936), daughter of the influential zoologist Giambattista Grassi (1854–1925) and the German-born campaigner for women’s rights Maria Koenen Grassi (1857–1943), founded FILDIS (*Federazione Italiana Laureate Diplomate Istituti Superiori*) as an offspring of the *International Federation of University Women* (IFUW), launched in 1919 (Oertzen, 2014). FILDIS instigated some noteworthy campaigns in support of university women, yet counted only very few members from Grassi’s personal circle among its members and was closed in 1935 by the Fascist regime (Taricone, 1992). The IFUW, too, attracted very few Italian women: in 1929 only ten (2.3%) and in 1932 only nine (1.6%) of all its members were Italian (IFUW, 1929, 1932). Not one of them was a life scientist. One factor behind the apparent individualism of Italian women biologists may have been a fear to be perceived as aggressive and engaged in political campaigning rather than thorough laboratory work. Another factor was that the Italian women’s associations initially offered little support for women with academic aspirations. On the contrary, pathbreaking graduates like Anna Kulicioff (1857–1925) and Ernestina Paper disdained the pursuit of scientific careers by women and rather wanted women doctors to fight for people’s health (Passione, 2007, p. 10).

Therefore, women who wanted an academic career probably prioritised finding male mentors. As we have seen, most Italian science departments were open to female students and assistants, and the departments of Cattani and Monti, in Bologna and Pavia respectively, were among the most inclusive. Two of Monti’s later opponents, Giambattista Grassi and Camillo Golgi (1843–1926), were widely known to encourage women to work in their laboratories, too. Still, these mentors in many cases limited their praise for and support of female colleagues to the role of subordinate assistants. Margaret Rossiter calls this phenomenon the ‘harem effect’, i.e., powerful male scientists like to surround themselves with female collaborators, because women often receive less pay, show less personal ambition and hence create less conflict within the research group (Rossiter, 1980). Maria Bakunin served as an assistant in her husband Agostino Oglialoro-Todaro’s (1847–1923) Chemical Institute for seventeen years before she became a professor (Colella, 2014, pp. 146–147). Agrarian entomologist Anna Foà (1876–1944) became a tenured professor only in 1924, one year before the death of her mentor Giambattista Grassi, whom she served as a teaching and research assistant for more than a quarter-century (Linguerri, 2007). Most of her publications were with Grassi and appeared almost exclusively in in-house journals. At a time when the career strategy of most Italian junior researchers included publication of notes and essays in prominent foreign journals (Dröscher, 2011), Foà’s preference for the local journal points to her acceptance of Grassi’s authority. The case of Cattani is similar. As mentioned above, she was very prolific; and yet, eighteen of her twenty-three publications on cholera and tetanus were co-authored by Guido Tizzoni (1853–1932). Apart from the ‘harem effect’, Cattani was probably also a victim of the ‘Mathilda effect’, i.e., the repeated attribution of the results and achievements of female researchers to their male colleagues (Lincoln et al., 2012; Rossiter, 1993). Tizzoni torpedoed her candidature in a professorship competition (Passione, 2007, pp. 16–19), but started with her a fruitful collaboration on tetanus, for which he was nominated as a Nobel candidate, whereas Cattani’s contribution still awaits recognition (Zannotti, 1988). After her move to Imola, she declared that she enjoyed her autonomy, yet she published no further papers.

Another reason why Italian pioneer women often were alone in their fight for institutional recognition lies in their research topics. Many female life scientists were active in emerging research areas, where they (initially) faced less male competition. However, these areas were still weak institutionally and lacked powerful lobbies that would fight for new chairs and institutes. Today, for instance, we regard Giuseppina Cattani’s research as cutting-edge, but it was not before 1907, hence ten years after she left university, that the discipline of bacteriology obtained its first associate professorship and a proper academic research institute in Italy.^1^ Rina Monti, too, experienced the institutional disadvantage of pioneering new research fields. Her neuroanatomical research in Camillo Golgi’s famous laboratory, attracted international awards and praise. Not so her work on fresh water ecology. Today, she is recognized as an international pioneer of limnology, but in the first decade of the twentieth century, the members of appointments commissions found it easy to belittle the scientific relevance of her results. Moreover, Giambattista Grassi, who was a member of almost all commissions for chairs of zoology, had himself taken up hydrobiology, yet he conceived of the field rather narrowly as applied pisciculture and not as an interdisciplinary and ecosystemic investigation like Rina Monti. Therefore, Grassi missed no opportunity to downplay her limnological works as superficial (Dröscher, 2007, pp. 139–142).

The third obstacle in Monti’s and Cattani’s way was Italy’s peculiar institutional context. Italian university professors were recruited principally by means of national competitions. At least from the 1880s, a handful of established leaders exerted a controlling influence on appointments in their disciplines (Dröscher, 2002, pp. 32–33; Dröscher, 2017, pp. 110–111). Candidates without significant political backing had almost no chance. Around the turn of the century, Cattani’s mentor Tizzoni was indeed influential, yet not as influential as Giulio Bizzozero (1846–1901), who placed nine of his students on university chairs of general pathology. Most of these young pathologists were excellent researchers, but the less brilliant ones made it anyway. Tizzoni, by contrast, as a member of the commission for the chair in Pisa, instead of promoting his pupil Cattani, induced her to withdraw her application. The experience of aforementioned paediatrician Angiola Borrino was similar. After losing her tenured chair in 1924, she was repeatedly ranked second behind candidates with much less experience but who were endorsed by one of the two rival schools of paediatrics that divided Italy’s chairs amongst themselves (Farnetani, 2018, pp. 12–16). Another example is botanist Eva Mameli Calvino (1887–1978), mother of the novelist Italo Calvino (1923–1985). In Pavia, she was student to the influential botanist Giovanni Briosi (1846–1919). When he died, Mameli lacked critical support during the crucial years of her early career. Luckily, she was protected by her brother Efisio (1875–1957)—who was twelve years her elder and a politically active chemist and university professor—and managed in 1927 to become the first Italian female professor of botany (in Cagliari), albeit the position was non-tenured and she retired after only two years owing to the rigidity of the university administration (Forneris & Marchi, 2004). Rina Monti also experienced the importance of political power. The sudden death of her mentor Pietro Pavesi (1844–1907) exposed her to the severe appraisals from Grassi, whose moves in pushing through his own students were on everyone’s lips (Dröscher, 2007, pp. 140–141).

Paola Govoni (2015, p. 76) argues that male Italian scientists around 1900 still did not “perceive the threat from women graduates in science as possible competitors”. The experiences of Cattani and Monti demonstrate otherwise. The pushback against rival women was always strong. In fact, Rina Monti’s winning move was to increase her political lobbying. She asked for support from famous scientists such as Ernst Haeckel (1834–1919) and Gustav Retzius (1842–1919) (Dröscher, 2007, pp. 139–141), yet she mostly relied on her elder brother Achille (1863–1937), professor of morbid anatomy and a powerful politician, and his circle. This group endorsed her and helped her to accomplish her goal by implementing a twofold strategy. On the one hand, they shrouded her feminility and stressed that she “worked like a man”. On the other hand, they played on the token of her femminility and argued that she was only overlooked because she was a women. Though not eschewing her feminility, marriage and motherhood, Rina Monti herself was keen to present herself as utterly serious and not given to any form of feminine vanity. In 1906, in the course of the campaign around the appointment to the zoological chair at the Museum of Natural History in Milan, Monti’s mentor Pietro Pavesi wrote a letter praising her energy and probity, vouching that “the care for her family does not distract her from her tireless activity” (Pavesi cit. in Canadelli, 2008, p. 160). Other supporters emphasized that in past competitions she had been overlooked for “extra-scientific reasons”. Monti herself wrote a letter expressing her hope that Milan, “the most civil city of Italy” would not object to the appointment of a women (ibid., pp. 142, 157–158). Yet, despite all efforts, Grassi, supported by Golgi, again succeeded in having one of his students come out on top, even though his curriculum vitae was far less remarkable. However, shortly after this the Minister of education called Rina Monti onto the chair in Sassari.

## The 1920s and 1930s: A new era?

The first appointments of Italian women to university professorships did not represent a rupture of male dominion in academia. On the contrary, until the middle of the twentieth century, women continued to be common—yet not equal—at the level of students and lower academic positions, but they were still an exception at the level of university professors. Even in the 2000s, the presence of women professors in Italy’s universities is still far from parity, even though the number of women students and women post-docs have surpassed those of male counterparts (Pironi, 2020, p. 164–166; Rettaroli, 2014). One circumstance that stood in the way of greater support for women in the Italian life sciences during 1920s and 1930s was the socio-cultural context. The period was profoundly different from the preceding liberalist era. After the turn of the century, humanist and conservative currents slowly regained the upper hand in public debate, resulting in growing scepticism towards science, and a notable reversal of opinion about the role of women in society. Even more dramatic were the consequences of the racial laws and the Second World War.

The cultural idealism of two rather different philosophers, namely, Benedetto Croce (1866–1952) and Giovanni Gentile (1875–1944), reveals that the new attitudes towards women and science were not confined to a specific political party. In the early 1920s, both men had discharged the office of Italian minister of education for a short period, implementing policies that hampered science and favoured humanistic education. Gentile saw teachers as symbols of authority and hence preferred them to be male. His Education Act of 1923 raised the university fees for women. On the one hand, he supported women’s education and created specific institutions for them, yet he excluded them from becoming lyceum teachers in the disciplines he considered to be the most important, i.e., history, philosophy, Latin and Italian. This measure, however, had a positive effect on the study of the natural sciences. In fact, women’s enrolment in university science courses increased, reaching a peak in the early 1920s (Govoni, 2015, p. 79). However, the proportion of female students in the student body as whole declined. Bologna, for instance, saw an increase of the number of female science students, but a decrease in their overall proportion from 37.5 percent in 1894–1908 to 25.3 percent in 1909–1919, and 10.3 percent in 1920–1931 (Giumanini, 2004).

Given the lack of a complete list of women working in Italian universities after 1915, the official data of two single years, 1931 and 1939, may substantiate my thesis. At the level of research staff, ninety-five (21.9%) women held positions as assistants and technicians in the Italian academic science departments in 1931. This represents about one fourth (25.6%) of the total if we limit our view to the biological institutes (Ministero, 1931). Among these, the zoological laboratories of Paolo Enriques (1878–1932) in Bologna and Padua, and of Cesare Artom in Pavia had a particularly high share of female researchers (Volpone, 2012, pp. 221–223 and 227). During the 1910s and 1920s, of the sixteen collaborators in Enriques’s cytogenetic investigations, nine were women: Anna Luisa Valenti, Anna Meneghini, Rosa Urbinati, Angelina Buzzoni, Bianca Del Bianco, Jolanda Deschmann, Lina Moro, Lucia Musconi, Fausta Bertolini (1894–1966), and Anita Vecchi (1893–1953). In Pavia, four out of the five collaborators in Artom’s chromosome studies were women: Elsa Ravetta, Franca Cavallina, Emilia Stella (1909–1994), and Ida Branchini Scatizzi (b. 1907). Yet, few of them succeeded in pursuing a notable academic career. After 1931, Ida Scatizzi gave lessons in agricultural entomology and in genetics and race research; in 1938, Anita Vecchi became ordinary professor of zooculture; and in 1953, Emilia Stella, daughter of Rina Monti, became associate professor of zoology in Rome.

Among the higher ranks, the number of women remained very small. In 1915, the female private lecturers of biological subjects numbered a mere ten out of eighty-six (11.6%). As late as 1931, only four women were officially professors: Rina Monti and Anna Foà were tenured; Anita Vecchi and Concettina Scordìa were temporary professors in Bologna and Messina respectively.^2^ Eight years later, the share of female assistants and technicians in the biological disciplines had grown to 33.5 percent and that of female private lecturers to 30.6 percent. Yet, the number of professors only increased from four in 1931 to five in 1939, none of them was tenured: Scordìa in Messina, Giuseppina Zannoni taught plant pathology in Genoa, Anita Vecchi gave a course in hydrobiology in Bologna, Carmina Manunta in general biology in Pavia, and Valeria Bambacioni (1895–1972) in genetics in Rome. Apart from these five, Anita Vecchi had become associate professor in the Agricultural department in Bologna (Ministero, 1939).

A systematic survey of the careers of Italian women who undertook research at the Zoological Station Anton Dohrn in Naples provides some useful data about new generations of female postgraduates. Around 1900, a research stint at Dohrn’s private and international research institution (Groeben, 2020) was an almost indispensable career step for Italian zoologists (Dröscher, 1996, pp. 95–96). During the period 1876–1942, 179 women from many countries went on 336 research sojourns at the station (Dröscher, 2022). This is a remarkable number considering that the Neapolitan municipality gave Dohrn the permission to found his private research institution with the proviso that “it is explicitly forbidden for women to stay here”.^3^ However, the number is small if compared to that of male researchers, who made up 88.5 percent of the research visits. Italian women made 130 visits. From 1908 onwards, Italian women were common guests at the *Stazione*, and during the period 1924–1942, their visits reached a remarkable 6.6 per year.

However, whereas many of the American and several of the German and British women visitors had important careers, the Italians did not. Most of the Italian female researchers at the Naples station, such as Bice Ferrari (b. 1872) and Ofelia Poso (b. 1881), completed the experimental part of their dissertations and then became schoolteachers. Others continued their research for a considerable period, but did not succeed in obtaining academic positions. Examples are Isabella Iroso, who spent seventeen years (1911–1928) at Dohrn’s working tables, the aforementioned Fausta Bertolini, who used the facilities in Naples between 1928 and 1937, and Beatrice Torelli. Torelli started to work at the *Stazione* in 1921 and then without interruption from 1924 to 1970 as assistant of Giuseppe Colosi (1892–1975), publishing numerous papers on crustacean and then cnidarian biology (Innocenti, 2007). Italian women did not have a privileged access like their American colleagues, who from 1898 to 1933 ran a special American Women’s Table (Sloan, 1978), but were sent by their institutions. Many directors of these institutions regarded the research facilities at the *Stazione* as an extension of their own university laboratories. Therefore, the above-described master-student dynamics were also at work in Naples, and many women researching at Dohrn’s institute continued to be the subordinate collaborators of Italian professors.

A handful of ‘Dohrnian women’ had some kind of professional career in science and was known for their expertise. However, these women carried on investigative enterprises as partners in creative couples. Collaborative and mutually inspiring partnerships, as those studied mainly in the biological sciences in the United States, Great Britain, and Germany (e.g. Abir-Am & Outram, 1987; Pycior, Slack & Abir-Am 1996; Richmond, 2006; Richmond, 2012; Satzinger, 2008; Velasco Martín, 2020; Vogt, 2000), existed in Italy, too. Examples include Ines De Stefani (1895–1941) and Giuseppina Benazzi Lentati (1905–1994). Ines De Stefani married the aforementioned professor of zoology Giuseppe Colosi. Collaborating and co-authoring papers with her husband, Ines abandoned her initial geological interests and devoted herself to zoology. Her work was extremely valuable in providing the empirical data to support his theories (Colosi, 1941), yet she also published in her own name, in particular on the systematics of crabs and on the fluid balance of terrestrial animals. She died of tuberculosis at the young age of forty-six.

Giuseppina Lentati’s collaboration with her husband Mario Benazzi (1902–1997) lasted almost seventy years. According to her student Giorgio Mancino, she never had an ambition to have a career of her own (Mancino, 1996). She knew Benazzi as a student at the Anatomical Institute of Turin, and followed him to Sassari, Siena and finally Pisa. Together, they made significant contributions to the emerging modern synthesis, in particular in cytogenetics, biogeography, population and developmental genetics and evolution, and won international recognition. Among numerous publications, in 1976, they co-authored a volume on flatworms (Platyhelminthes) in Jon Bernard’s prestigious series *Animal cytogenetics*. Although the scientific community regarded Giuseppina Lentati as working symbiotically with her husband, she published almost half of her 56 papers in her own name.

A “non-Dohrnian” example of Italian creative couples is Eva Mameli Calvino. After the birth of her second son, the geologist-to-be Floriano Calvino (1927–1988), she availed herself of the opportunity to work (with a salary) at her husband Mario Calvino’s (1875–1951) Station of Experimental Floriculture in Sanremo, near Nice. She published more than 200 papers, contributing to the development of floriculture, a branch of considerable economic importance in the region (Forneris & Marchi, 2004).

Only in-depth analysis of the individual cases can reveal for how many of these women the close collaboration with their marriage partners represented mutual support, a unique opportunity to continue high-level research that would otherwise have been impossible, or scientifically unjust subordination and condemnation to invisibility. Likewise, future analysis needs to assess the effect of such relationships on the gender structure of the couple’s research staff, and on the social functioning and moral economy of their laboratories.

Hence, for many years Rina Monti’s ordinary professorship remained an exception in the Italian academic life sciences, as did that of Maria Bakunin in chemistry. Their stories as well as the later ones of Foà and Mameli rather seem to be a continuation of the Italian tradition of exceptional women, who were endorsed by influential men and thus ‘allowed’ to fill important positions as long as they remained departures from the rule. Yet, by the 1930s, we find several differences. Firstly, the few female directors of institutes and laboratories, such as Foà and Monti, trained many young women, who later themselves pursued academic careers. Secondly, the number of women with university degrees and assistantships and therefore academic pressure from female biologists grew steadily. The increasing discrepancy between the number of women in the lower ranks and the small number among the higher ranks became unmissable. Even women who did not get temporary or tenured professorships contributed to an increase in women scientists working in the institutes and laboratories as graduates, interns, assistants and private lecturers, proving through collaborations and publications that the value of female researchers was not limited to a handful of exceptional women. Thirdly, alongside the classical university chairs, the twentieth-century life sciences offered several other new opportunities to work as professional scientists, in particular in institutions of applied biology.

## Bypassing the obstacles: the Italian way to female professorship

Benito Mussolini’s (1883–1945) attitude towards science was ambiguous (Benadusi, 2011). While the fascist regime fiercely opposed the secular, liberalist and rational aspects of science as a philosophy, it exalted the myth of science as a source of technological progress and national grandeur and autarchy. Anti-positivist proclamations did not prevent him from promoting institutions devoted to applied science and modern economic necessities. Therefore, paradoxically, just in this anti-science and anti-femminist period, we can observe the coming into being of new opportunities for women scientists. If the prestigious chairs of Italian universities continued to be unattainable for women, appealing options opened up with the foundation of research institutions of applied biology. In 1934, 54 experimental agricultural stations or laboratories and fourteen zootechnical institutes with several dozens of associated practical facilities operated throughout the peninsula. During the next years, this number grew further, followed by academic institutionalisation between 1940s-1960s. Numerous new chairs were established at the science, medical and agricultural departments (Volpone, 2012 pp. 89–100).

From today’s vantage point, it appears that research careers in peripheral institutes turned out to be the best way for Italian women to finally gain university chairs. Several of the new institutes focused on applied biology, such as Mameli Calvino’s Station of Experimental Floriculture. For Anita Vecchi, who we met earlier, this was an indirect path to professorship. She was a pioneer of the science of zooculture and animal husbandry in Italy and founder of the National Institute for Beekeeping and of the Provincial Station of Aviculture in Bologna. In 1922, she started to teach zooculture at the Department of Agriculture, the first such course in Italy. In 1938, this position was transformed into an associate professorship. Her successor in 1953 was another woman: Ida Giavarini (1908–1996).

The example of Rina Monti demonstrates that the decision to enter emerging research fields could mean double the disadvantage: of being a women and of being a disciple of a not yet wholly recognized discipline. On the other hand, innovative fields and new institutions brought more freedom to develop one’s own research style and topics, to work at some distance from academic rivalries, and ultimately to attain fame as a pioneer of a new discipline. The best known example is surely the physician and educator Maria Montessori. Despite much adversity, her intellectual autonomy soon paid off and her innovative neuropsychiatric and pedagogic method of early childhood education came to be internationally celebrated (Babini & Lama, 2000).

Perhaps the three most prominent disciplines in which Italian women scientists made their way were hydrobiology and limnology, entomology and cytogenetics. Monti, indeed, became a central figure of early-twentieth-century limnology. Not only did she contribute to founding the field, but she also played a crucial role in developing it as an ecological science, investigating not merely flora and fauna or hydrographical-chemical properties, but the reciprocal effects of animate and inanimate parts of the environment (Elster, 1974, p. 7). Moreover, her school trained a line of well-known women limnologists. Her student Livia Pirocchi (1909–1985) also promoted Monti’s holistic approach and combined it with modern genetic techniques to study the dynamic development of natural populations. Pirocchi became the ‘soul’ of the Italian Institute for Hydrobiology in Pallanza, a private research institute founded in 1937 on the southern shores of the Lake Maggiore that soon attracted illustrious international visiting scientists (Edmondson & Edmondson, 1990). During the war, she run a small clandestine refuge with her future husband Vittorio Tonolli (1913–1967), molecular biologist Adriano Buzzati Traverso (1913–1983) and population geneticist Luigi Luca Cavalli Sforza (1922–2018). In 1967, Pirocchi succeeded her husband as director of the Institute and promoted its incorporation into the Italian National Research Council (CNR). She published 79 papers and books.

Other women hydrobiologists included Anita Vecchi, Concettina Scordìa, Monti’s daughter Emilia Stella, and Ester Taramelli (1931–1990). Vecchi gave lessons in hydrobiology and pisciculture in Bologna from 1936, Scordìa became associate professor in the 1940s and directed the Institute of Hydrobiology and Pisciculture in Messina, Stella became associate professor of zoology in Rome in 1953, and Taramelli became associate professor of biological oceanography in Rome in 1983 (Crucitti & Bubbico, 2020, pp. 105–111).

A second research field that soon assumed a marked feminine character was applied entomology. From 1905 on, Anna Foà deployed her knowledge of insects at the Royal Antiphiloxeric Observatory at Fauglia near Pisa. Here, she worked under the guide of Giambattista Grassi and trained a new generation of male and female entomologists (Linguerri, 2007, pp. 171–172). In 1921, she became professor of sericulture at the Royal Agricultural School of Portici near Naples, moving in 1924 to a newly established chair at the University of Naples. Another of Grassi’s assistants, Lidia La Face (b. 1891), in 1923 became a member of the scientific staff of the Experimental Station for Antimalarial Research and then of the Antimalarial Laboratory of the Ministry of Health in Rome (Patuelli, 2012). Four other eminent women entomologists were Amelia Tonon (1899–1960) in Padua, Enrica Calabresi (1891–1944) in Florence and Pisa, and Marta Grandi (1915–2005) and Maria Matilde Principi (1915–2017) in Bologna. Tonon worked at the Experimental Station of Sericulture from 1923 to 1960, Calabresi became adjunct professor of entomology at the Department of agriculture in Pisa in 1936, Grandi published 46 papers on insects, especially mayflies (Ephemroptera), but did not have a university career, and her colleague Principi became professor of Entomology in Bologna in 1958.

The third prominent area was cytogenetics. In line with developments in other countries (see e.g. Deichmann, 2008; Richmond, 2007; Stamhuis & Monsen, 2007), during the early period of Italian genetics, many research directors were pleased to find skilled female research staff for a field that was still not sufficiently attractive to ambitious young male students, thus indirectly clearing the way for at least three distinguished careers: Luisa Gianferrari, Valeria Bambacioni and Eleonora Francini. Luisa Gianferrari (1890–1977), a trained biologist, focused on human genetic diseases, Mendelian statistics, and eugenics. She studied natural sciences in Innsbruck but graduated in 1918 from Bologna, then moved to Rome and finally Milan, where she gave courses, from 1924 to 1933, in general biology and in experimental embryology and genetics. In 1940, she founded the Study Center in Human Genetics with the purpose of creating a national genetic register of the Italian population. After the war, she continued to promote eugenics, but turned from praising to condemning the Nazi legislation (Cassata, 2011, pp. 272–284 and 309–323). In 1950, she obtained the first official chair of human genetics in Italy.

Another pioneer of genetics in Italy was the aforementioned Valeria Mezzetti Bambacioni, student of the plant physiological school of Romoaldo Pirotta (1853–1936) in Rome. She gained international celebrity thanks to a series of studies carried out in the late 1920s. Combining her embryological and cytogenetic skillsets, she clarified in detail the mechanism of transitory polyploidy (cells possessing temporarily more than two sets of chromosomes) during the gametogenesis of the Liliaceae *Fritillaria* and then of other species. Her Italian colleagues initially downplayed her results, until foreign botanists recognized it and named it the ‘Bambacioni phenomenon’ or ‘Bambacioni effect’ (Mazzolani, 1978). She started to teach genetics in 1937 while she was still an assistant in Rome and continued these lectures a few years later as a temporary professor of botany and genetics at the University of Messina and from 1948 at the Royal Agricultural School in Portici, near Naples.

A similar cytogenetic-embryological approach also distinguished the early research on polyploidy and chromosomal variability in plant cells carried out by Eleonora Francini Corti (1904–1984), who then switched to ecology and phytogeography. In 1939, she was appointed to the chair of botany at the Department of Agriculture in Bari, creating a functional institute. In 1961, she joined her husband, forestry scientist Roberto Corti (1909–1986), at the Science department in Florence, leaving the position in Bari to her student Albina Messeri (1904–1972).

This demonstrates that, the prevailing anti-feminist and anti-science policies notwithstanding, several women managed to launch prominent careers in science during the 1920s and 1930s. However, the negative pressures during this period, in particular due to the racial laws of 1938, were far greater. Ninety-six Jewish university professors, about two hundred assistants, researchers and private lecturers, countless students and graduates were expelled from the universities—among them many women, such as the botanists Pierina Scaramella (1906–1996) and Gina Luzzatto (b. 1904), biochemist Ada Bolaffi (1900–1980), comparative anatomist Isabella Lattes Coifmann (1912–2006), pharmacologist Angelina Levi (1892–1975), but also schoolteachers like Fausta Bertolini. Historian Raffaella Simili thinks that many, already working in subordinate or informal positions, continued during these dark years to live in Italy with false identities and to carry out research privately (Simili, 2010). Rita Levi Montalcini, for instance, worked as Rita Lupani. Isabella Lattes Coifmann began to publish popular science essays under a pseudonym, and continued to do so with great success after the war.

Not all of them reappeared after 1944–45. Anna Foà, who was forced to leave her university professorship and cease her membership in the Neapolitan Society of Naturalists, probably continued to live in Italy under a false name, but died around July 1 or 2, 1944. Enrica Calabresi had been assistant to zoologist Angelo Senna (1866–1952) in Florence from 1914. In 1932, she resigned from university, when she was forced to vacate her position as ‘first assistant’ for a younger colleague, the known fascist count Lodovico Di Caporiacco (1900–1951), a decision she saw as a great injustice (Ciampi, 2006). In 1936, however, she became adjunct professor of entomology in the Department of Agriculture in Pisa. The racial laws again forced her to leave university, but she continued to teach the Jewish children who were barred from attending school in Florence until her arrest in January 1944. To escape deportation to Auschwitz she poisoned herself to death.

Other women, not all Jewish, left Italy. The most famous emigrant was Rita Levi Montalcini. She continued to maintain strong ties to Italian science, yet the epicentre of her research moved to the United States. In 1939, botanist Ada Silvia Colla (1902–1989) immigrated to Argentina, where she founded the country’s first institute of plant physiology (Luciano & Roero, 2008, pp. 106–115). Zoologist Maria Romano (b. 1913) and her husband Giorgio Schreiber (1905–1977) immigrated to Brazil, where he directed the Laboratory of Cytology and Genetics in Sao Paolo and then Belo Horizonte. In 1947, embryologist Eugenia Tamini followed her husband, geologist Alberto Parodi (1907–1999), to Peru, where she taught comparative anatomy and microbiology at the University San Augustìn in Arequipa.

## Conclusion

From the 1900s, women became a common presence in Italian university lecture halls and, in smaller numbers, among the laboratory and institute staffs, in particular in the life sciences. However, at least until the mid-twentieth century, there were only exceptional cases of women winning appointments to a university professorship. The data assembled in this paper demonstrate the ongoing tension between the Italian tradition of universities being relatively open to women and the rarity of female professors. Women like Rina Monti and Maria Bakunin succeeded in gaining high-ranking university positions thanks to endorsement by politically powerful male mentors, mainly husbands or brothers. The contemporaneous examples of Giuseppina Cattani and Anna Foà, on the other hand, show that male professors continued to regard women scientists mainly as (skilled) subordinate collaborators. Only slowly, the increasing presence of women at the lower ranks of the academic hierarchy and their demonstrated ability to produce valuable scientific results began to exert more pressure for institutional recognition. During the 1920s and 1930s, the situation of Italian women life scientists changed significantly, but remained complex. On the one hand, the general socio-political context became increasingly anti-scientific and anti-feminist; on the other hand, emerging disciplines and newly founded peripheral institutes of applied science provided new career opportunities that were embraced by many women. The research fields of hydrobiology, entomology and cytogenetics were of particular importance in women’s path towards equality.

## Footnotes

1 Both were instituted in Naples. In other Italian universities, bacteriology was taught from 1906 onwards by contract teachers (*incarico*).

2 Other female tenured professors in the science departments were the two mathematicians Margherita Beloch Piazzolla (1879–1976) in Ferrara and Pia Nalli (1886–1964) in Catania, and the physicist Rita Brunetti (1890–1942) in Cagliari. Anna Fiori (b. 1902) was temporary professor of Palaeontology in Bologna, Maria Kahanovicz of Physics in Sassari.

3 I am indebted to Christian Groeben for this information.
